# Spatial scaling of plant and bird diversity from 50 to 10,000 ha in a lowland tropical rainforest

**DOI:** 10.1007/s00442-021-04925-8

**Published:** 2021-05-05

**Authors:** Richard J. Hazell, Kryštof Chmel, Jan Riegert, Luda Paul, Brus Isua, Graham S. Kaina, Pavel Fibich, Kenneth Molem, Alan J. A. Stewart, Mika R. Peck, George D. Weiblen, Vojtech Novotny

**Affiliations:** 1 Department of Evolution, Behaviour and Environment, School of Life Sciences, University of Sussex, Brighton, BN1 9QG UK; 2 Biology Centre, Institute of Entomology, Czech Academy of Sciences, Branišovská 1760, 370 05 Ceske Budejovice, Czech Republic; 3 Faculty of Science, Charles University, Viničná 7, 128 44 Prague, Czech Republic; 4 Faculty of Science, University of South Bohemia, Branišovská 31, 370 05 Ceske Budejovice, Czech Republic; 5 New Guinea Binatang Research Centre, Madang, Madang Papua New Guinea; 6 Papua New Guinea Forest Research Institute, Lae, Morobe Papua New Guinea; 7 Department of Plant Biology, University of Minnesota, Saint Paul, MN 55108 USA

**Keywords:** Beta-diversity, Community composition, ForestGEO plot, Rarefaction, Species richness

## Abstract

While there are numerous studies of diversity patterns both within local communities and at regional scales, the intermediate scale of tens to thousands of km^2^ is often neglected. Here we present detailed local data on plant communities (using 20 × 20 m plots) and bird communities (using point counts) for a 50 ha ForestGEO plot in lowland rainforest at Wanang, Papua New Guinea. We compare these local diversity patterns with those documented in the surrounding 10,000 ha of lowland rainforest. Woody plant species richness was lower within 50 ha (88% of 10,000 ha richness), even when both were surveyed with identical sampling effort. In contrast, bird communities exhibited identical species accumulation patterns at both spatial scales. Similarity in species composition (Chao-Jaccard) remained constant while similarity in dominance structure (Bray–Curtis) decreased with increased distance between samples across the range from < 1 to 13.8 km for both plant and bird communities. The similarity decay was more rapid in plants, but in both cases was slow. The results indicate low to zero beta-diversity at the spatial scale represented here, particularly for birds but also for woody plants. A 50 ha plot provided a highly accurate representation of broader-scale diversity and community composition within 10,000 ha for birds, and a relatively good representation for woody plants. This suggests potential for wider generalization of data from ForestGEO plots which are almost always locally unreplicated, at least for those in lowland tropical forest.

**Supplementary Information:**

The online version contains supplementary material available at 10.1007/s00442-021-04925-8.

## Introduction

Diversity patterns are scale-dependent (Willis and Whittaker 2002; Rahbek 2005; Jackson and Fahrig 2015), and as such the magnitude of diversity is likely to vary with geographic scale (Crawley and Harral 2001; Rahbek 2005). For example, Lennon et al. (2001) found that patterns of British bird species richness at a scale of 10 km were statistically unrelated to those at a scale of 90 km. Scale may be measured in terms of grain, i.e. the size of individual observation units (as in the above case), or extent, i.e. the total area covered by the observations within a survey (Wiens 1989). The manipulation of either of these aspects is likely to affect the outcomes of diversity estimates in different ways (Nekola and White 1999). With this in mind, the extent to which diversity estimates can reasonably be extrapolated to areas larger than the area sampled is limited (Colwell and Coddington 1994). Any such inferences require an understanding of taxon-specific patterns of alpha-diversity in local communities and beta-diversity, i.e. turnover in species composition between sites.

Both alpha- and beta-diversity are likely driven by a variety of ecological mechanisms, from niche-based processes such as environmental filtering and biotic interactions to local stochastic processes and spatial factors such as dispersal limitation, with little consensus over which processes are dominant in structuring communities (Macarthur and Levins 1967; Vandermeer 1972; Hubbell 2001; Veech and Crist 2007; Rosindell et al. 2011; Myers et al. 2013; Yang et al. 2015). In reality it is likely that the relative importance of the different mechanisms shaping both alpha- and beta-diversity varies according to the spatial scale at which analyses are conducted (Rahbek and Graves 2001; De Cáceres et al. 2012; Barton et al. 2013; Chase 2014; Melchior et al. 2017). In a meta-analysis of plant and animal species richness gradients across scales, Field et al. (2009) found that climate and productivity were the best predictors of richness at the largest grain and extent sizes, while area was strongest at small (< 10 km^2^) and biotic interactions at intermediate scales (10–500 km^2^). In addition, the importance of different processes may vary between taxa. Different taxa may show contrasting diversity patterns across scales (Qian 2009; Zellweger et al. 2017) due to variation in energy sources and dispersal ability, as well as the scale at which habitats are perceived and niches defined (Barton et al. 2013).

Plants and birds are both important components of tropical rainforest systems, but the extent to which diversity estimates made at one scale translate to another may differ between them. For example, plant distribution may be influenced locally by fine-grained environmental characteristics such as soil composition which do not directly affect birds (Idárraga et al. 2016). Nevertheless, bird community structure is clearly affected by variations in habitat structure created by plants themselves (Jankowski et al. 2013). Reif et al. (2008) found that patterns in bird community structure were primarily attributable to variation in habitat composition at small spatial scales (0.5 km), while dispersal limitation and historical factors became more important at a scale of 8 km. Studies focussing on plants show varying results, with some attributing large-scale patterns to broad-scale environmental gradients and finer-scale patterns to stochastic processes (Laliberté et al. 2008), and others showing apparently contrasting patterns (Freestone and Inouye 2006). Evidence for the processes structuring plant diversity in diverse uniform habitats such as lowland rainforests in the absence of clear climatic and environmental gradients is currently sparse, although some studies indicate that spatial processes such as dispersal limitation may predominate here at intermediate scales (Condit et al. 2002; Myers et al. 2013).

Forest dynamics plots provide an effective means of assessing long term changes in biodiversity patterns of vegetation mapped in great detail but on a relatively small spatial scale of 15–50 ha (Condit 1995). The Forest Global Earth Observatory (ForestGEO) now comprises a global network of such plots (Anderson-Teixeira et al. 2015). The comprehensive inventories of all woody plants ≥ 1 cm diameter at breast height (DBH) in these plots quantify plant species diversity in a standardised manner for forests across the tropics (Ashton 1995; Condit 1998). The detailed spatially explicit information on plants in forest dynamics plots presents an opportunity for complementary surveys of animal communities, including birds, which has not been used so far.

Interestingly, most of the ForestGEO plots lack complementary estimates of plant diversity for the surrounding wider areas of 10–100 km^2^, relying thus on extrapolation of species diversity patterns across wider spatial scales (Kochummen and LaFrankie 1990; Lee et al. 2002; Kenfack et al. 2007). Systematic quantitative surveys of biodiversity within tens to hundreds of km^2^ of relatively homogeneous habitats, such as lowland rainforests, are rare, compared to local community data on the one hand and data on regional floras and faunas on the other (e.g. Novotny et al. 2006; Basset et al. 2012). This reliance on extrapolation is presumably based on the long-recognised principle that diversity should increase with the size of area sampled (MacArthur and Wilson 1967). However, uniformity of patterns and processes across scales cannot be assumed (Wiens 1989; Scheiner et al. 2000). Scaling curves may vary from linear to more complex logistic patterns, depending on the range of scales involved and the characteristics of the environment and the taxa being studied (Barton et al. 2013). For example, increasing spatial extent may cause a rapid increase in diversity at fine scales due to stochastic variation in species occupancy patterns, which slows down at intermediate scales as the number of new species added relative to the regional pool decreases (the rate of this deceleration varying among taxa with an organism’s perception of habitat heterogeneity and its dispersal ability). At large scales, increases in diversity may start to accelerate again as separate biogeographic regions with distinct evolutionary histories are encompassed (Barton et al. 2013). This high potential for variation demonstrates how simple diversity extrapolations may produce results that vary considerably from reality.

This study aims to fill two gaps in rainforest biodiversity studies by (i) providing detailed spatially explicit data on community composition of birds within a 50 ha ForestGEO plot in Papua New Guinea (the Wanang forest dynamics plot, or FDP), thus matching similarly detailed information on plants, and (ii) examining the plant and bird data from the FDP in the context of the surrounding 10,000 ha of lowland rainforest (the Wanang Conservation Area, or WCA), focussing on the alpha- and beta-diversity patterns. Similarly to many lowland rainforests, the habitat surrounding the FDP is relatively uniform at the scale explored in this study, both in terms of elevation and climate (Paijmans 1976). Therefore we expect that environmental factors will play relatively little role in shaping alpha- and beta-diversity patterns of woody plants and birds at this scale, and that dispersal limitation will play a more important role (Myers et al. 2013). As birds are better dispersers than plants, our hypotheses thus primarily reflect the differences in dispersal limitation between the two taxa. We hypothesize that: (i) woody plant species richness will be higher in the WCA than in the FDP, while bird species richness in the FDP and WCA will be similar, and (ii) woody plant species dissimilarity will increase with increased distance between plots, while bird dissimilarity will show little or no change with increasing distance, indicating higher beta-diversity of plants than birds at the scale explored in this study.

By including data from both plants and birds and thus incorporating a broad trophic range, this study aims to provide a more complete picture of spatial diversity patterns than those produced by studies focussing on plants alone. Such studies currently comprise the vast majority of data from forest dynamics plots (e.g. Hubbell and Foster 1983; Condit et al. 1996; Plotkin et al. 2001; Lee et al. 2002; Volkov et al. 2005, 2009; Kenfack et al. 2007; Metz 2012; Chen et al. 2016). Indeed, although the nature of the data obtained by surveys of plant and bird communities using vegetation plots and bird point counts respectively is essentially identical, comprising the list of all individuals from a defined area often between 0.05 and 1 ha, the two taxa are rarely studied simultaneously (but see e.g. Schulze et al. 2004). This study is additionally the first to our knowledge to specifically assess the suitability of forest dynamics plots as a monitoring tool for assessing bird diversity. Finally, by keeping grain size constant between surveys this study specifically investigates the effect of spatial extent on the diversity patterns observed.

## Materials and methods

### Study site

**Fig. 1:**
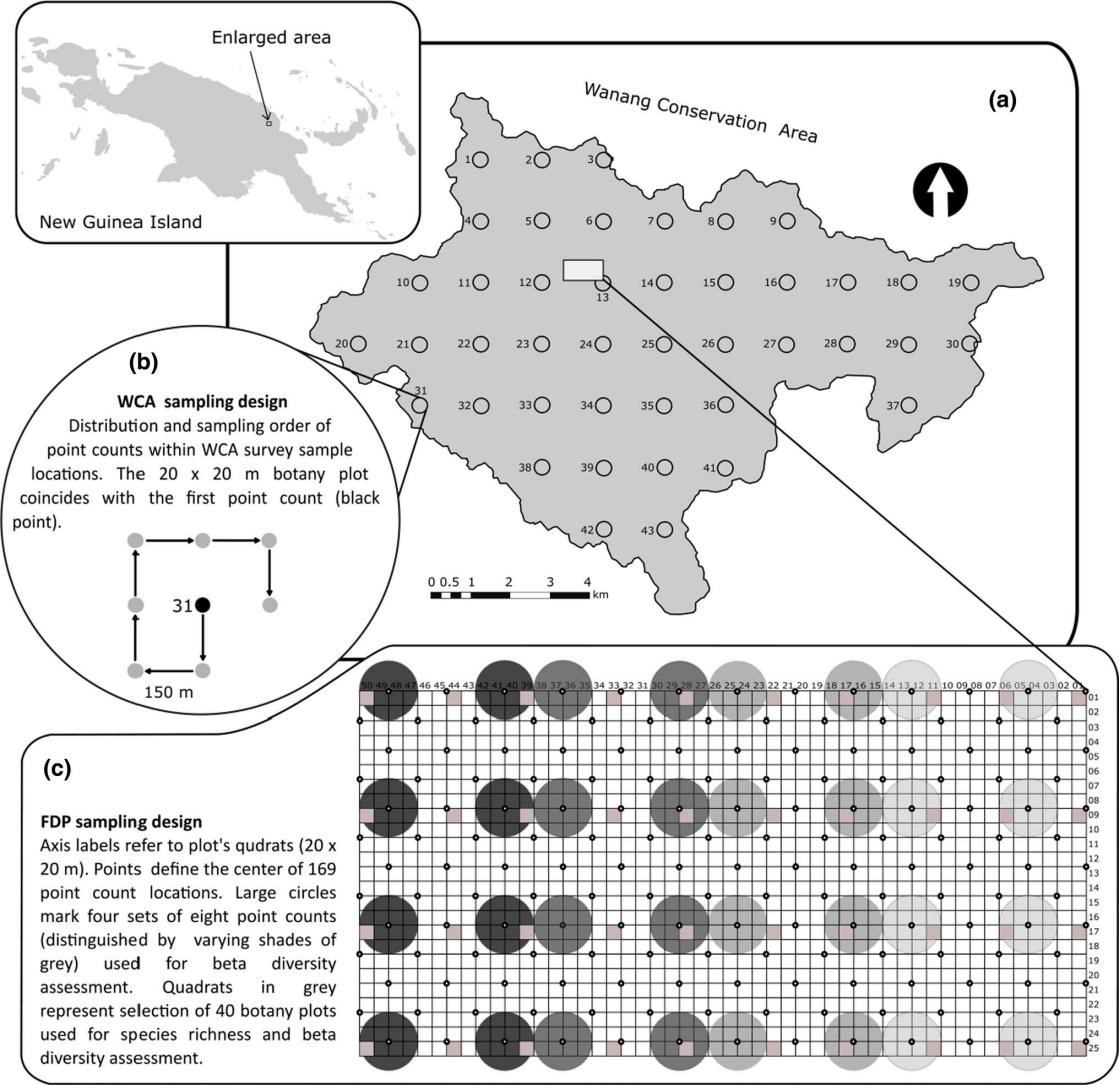
Map and diagrams showing **a** geographic location of Wanang Conservation Area (WCA) in New Guinea, **b** layout of woody plant and bird sampling locations in the WCA (circles) and **c** sampling design in the 50 ha Forest Dynamics Plot (FDP). One botany plot and eight point counts were sampled in each location of the WCA. Bird surveys at sampling locations 1, 20 and 36 were omitted due to logistical constraints

The Wanang Conservation Area (WCA) comprises 10,770 ha of primary lowland rainforest in the Middle Ramu region of Madang Province, northern Papua New Guinea. The forest is classified as tropical, wet mixed evergreen (Paijmans 1976). The climate, with an average temperature of 25.8 °C and annual precipitation of 4000 mm (Vincent et al. 2015), has a mild dry season from July to September. Although a lowland site, the topography is variable and comprises steep ridges separated by a network of streams and rivers. The sample sites range in elevation between 80 and 250 m above sea level, encompassing the full topographical range of the WCA. The 50 ha forest dynamics plot (FDP) is located centrally within the WCA (Fig. 1) and comprises a 1000 × 500 m rectangle divided into 1250 individual 20 × 20 m plots. Its location was selected in part to encompass as fully as possible the topographical range of the WCA, with elevation ranging from 90 to 180 m a.s.l.

### Vegetation survey

Plants in the FDP were surveyed using a standard methodology for ForestGEO plots (see Anderson-Teixeira et al. 2015). That is, all free-standing trees with DBH ≥ 1 cm were tagged, mapped, measured and identified to species level. Only woody plants ≥ 5 cm DBH were included in the present analysis. The WCA was surveyed by a regular grid comprising 43 sites 1.5 km apart, fitted to the conservation area using ArcGIS 10.02 (Fig. 1). Each sample location included a single 20 × 20 m plot, mirroring the sample design of the FDP. All plant stems with DBH ≥ 5 cm were measured and identified within each plot. Data collection took place from 4-Oct-2014 to 17-Dec-2014 in the WCA and from 2010 to 2012 in the FDP. In addition to species composition, each plot was characterized by canopy height, canopy closure, total tree basal area, number of stems and plot elevation. These variables were selected to enable characterisation of topographical heterogeneity between plots for both plants and birds (elevation), and of structural habitat heterogeneity between plots for birds (four vegetation characteristics). Canopy closure was assessed in matlab version 2015a (Mathworks 2015) by measuring the mean percentage cover of foliage in four canopy photos from each 20 × 20 m plot, using code developed by Korhonen and Heikkinen (2009). In the field, sample points were located using GPS (Garmin GPSmap 62 s).

### Bird survey

Bird surveys were based on point counts. The FDP was surveyed using a regular grid of 169 points separated by 80 m along the horizontal and vertical lines parallel to the plot boundaries (Fig. 1). Each point was used for one point count. Bird surveys in the WCA used the same 1.5 km grid as the vegetation surveys. Forty sample locations were sampled by eight point counts separated by 150 m, with the first point count being coterminous with the 20 × 20 m plot of the vegetation survey (Fig. 1). This gave a total of 320 individual point counts.

Point counts followed the same protocol for both sets of surveys. Counts took place between 06:00 and 10:30 am, lasted 10 min and started after an interval of a few minutes following arrival at each point to minimize the effects of disturbance caused by arrival (Bibby et al. 2000). All individual birds seen or heard were recorded together with an estimate of their distance from the observer. Only birds estimated to be within 40 m of the observers were included in the analysis. To minimize multiple counts of the same individual, multiple conspecifics were recorded only if the observer could be sure they were different individuals (e.g. two birds singing simultaneously). Point counts were always conducted by two observers, one of whom (LP) was present across all surveys to minimize the effects of observer bias. During each point count, an audio recording was made using an Olympus LS-5 Linear PCM digital recorder. This enabled later identification of misidentified or poorly heard individuals. Field work took place from 4-Oct-2014 to 31-Jan-2015 in the WCA and from 10-Feb-2015 to 6-Mar-2015 in the FDP.

### Data analysis

For both woody plants and birds, species richness for the FDP and WCA were compared using R package “iNEXT” for rarefaction and extrapolation of species richness (Hsieh et al. 2016). For plants, 40 20 × 20 m plots from within the FDP were selected to enable comparison of equivalent sampling effort with the WCA (Fig. 1). Sample-based rarefaction and extrapolation curves for both plant and bird datasets were created using Hill numbers (with *q* = 0), i.e. species richness unbiased by abundances of individual species (Chao et al. 2014). Sample-based rarefaction was chosen over individual-based since samples approximate more closely independent data points than do individuals and as such account better for natural levels of environmental heterogeneity present (Gotelli and Colwell 2001). A bootstrap method based on 999 random permutations of the data enabled the construction of confidence intervals and comparison of overlap at the maximum point for which sampling effort was equal (40 samples for plants; 169 samples for birds). In addition, the Chao2 richness estimator (Chao et al. 2005) was calculated for each dataset to give an estimated value of asymptotic species richness.

Plant beta-diversity was analysed using the 40 20 × 20 m plots from the FDP selected for the species richness analysis, providing a range of pairwise distances between plots from 0.1 to 1.12 km, in addition to the 43 20 × 20 m plots from the WCA, having pairwise distances ranging from 1.5 to 13.8 km (Fig. 1). For birds, the 40 individual sample locations combining eight point counts each were used as data points for the WCA. This data structure was mirrored in the FDP by creating four sets of eight adjacent point counts (spaced 160 m apart, Fig. 1) used as equivalents of the WCA sample locations. The Bray–Curtis dissimilarity and the Chao variant of the Jaccard dissimilarity indices were employed for beta-diversity analyses using the “vegan” package in R (Oksanen et al. 2018). The choice of these indices enabled us to explore dissimilarities based on species composition (Chao-Jaccard) or where the species abundance is also taken into account (Bray–Curtis) (Chao et al. 2005).

For analysis of both plant and bird beta-diversity across space, data from the FDP and WCA were pooled. The community dissimilarity was correlated with distance by plotting the Bray–Curtis and Chao-Jaccard matrices against a between-site distance matrix, using a Mantel test (9999 permutations) with Pearson’s correlations. In addition to the analysis on the pooled datasets, we compared variability in dissimilarity between sample locations in each surveyed area (the FDP versus the larger WCA) to reveal whether communities are more similar within the smaller sampling area. Differences in dissimilarity between the FDP and WCA were tested using a permutational MANOVA with 999 permutations provided by function *adonis* in the “vegan” package (Oksanen et al. 2018).

To determine the relative importance of spatial and environmental variables in determining woody plant and bird community composition, we used canoco 5 (Smilauer and Leps 2014) to perform a Principal Coordinates of Neighbouring Matrices (PCNM) analysis using Canonical Correspondence Analysis (CCA) for the woody plant dataset and Redundancy Analysis (RDA) with forward selection for the bird community dataset. Based on the length of the first Detrended Correspondence Analysis axis, canoco 5 recommends either a unimodal (CCA) or linear (RDA) method. The PCNM approach enabled us to separate the effect of space predictors (represented by spatial eigenfunctions corresponding to spatial relationships among the sampling sites) from the effect of primary (environmental) predictors (Legendre and Legendre 2012). The analysis included nine steps: primary predictor test, primary predictor selection by CCA, principal coordinate analysis (PCoA), PCNM for all predictors, PCNM selection, spatial effects analysis, primary predictor effects analysis, joint effects analysis and removal of spatial effects (Smilauer and Leps 2014). Elevation of vegetation plots was tested as the environmental primary predictor of woody plant composition. In the case of bird community composition, we included the following potential predictors in the analysis: canopy closure, tree basal area, total DBH of small (5–10 cm DBH) and large stems (> 40 cm DBH), diversity of trees (Simpson Index) and elevation of sampling location. Elevation was averaged over eight sample points for a given sampling location.

## Results

### Species richness of woody plant community

**Fig. 2:**
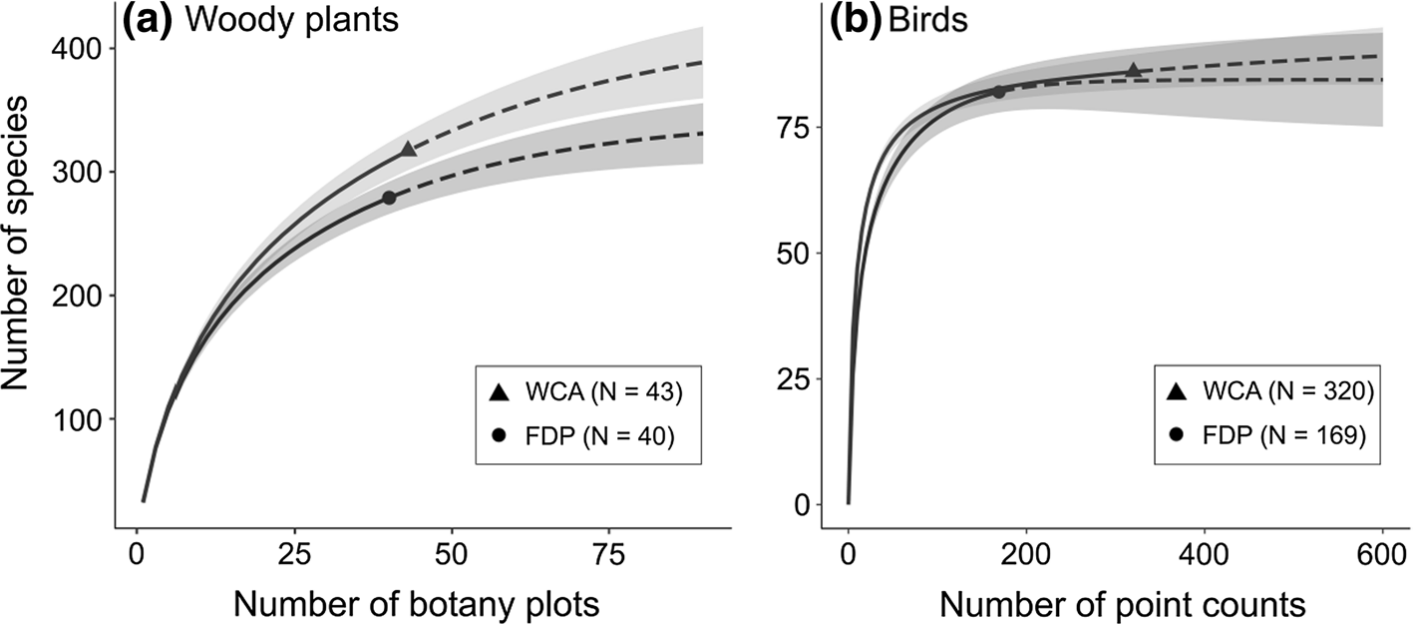
Species richness rarefaction curves for **a** woody plants and **b** birds in the WCA and FDP. Solid lines show interpolated rarefaction curves. Dashed lines represent extrapolated rarefactions exceeding our sampling effort. Shaded areas represent ± 95% confidence intervals. Species accumulation was calculated for woody plants from 20 m x 20 m plots (*N* = 43 and 40 for WCA and FDP, respectively) and for birds from point counts (*N* = 320 and 169 for WCA and FDP, respectively)

**Fig. 3:**
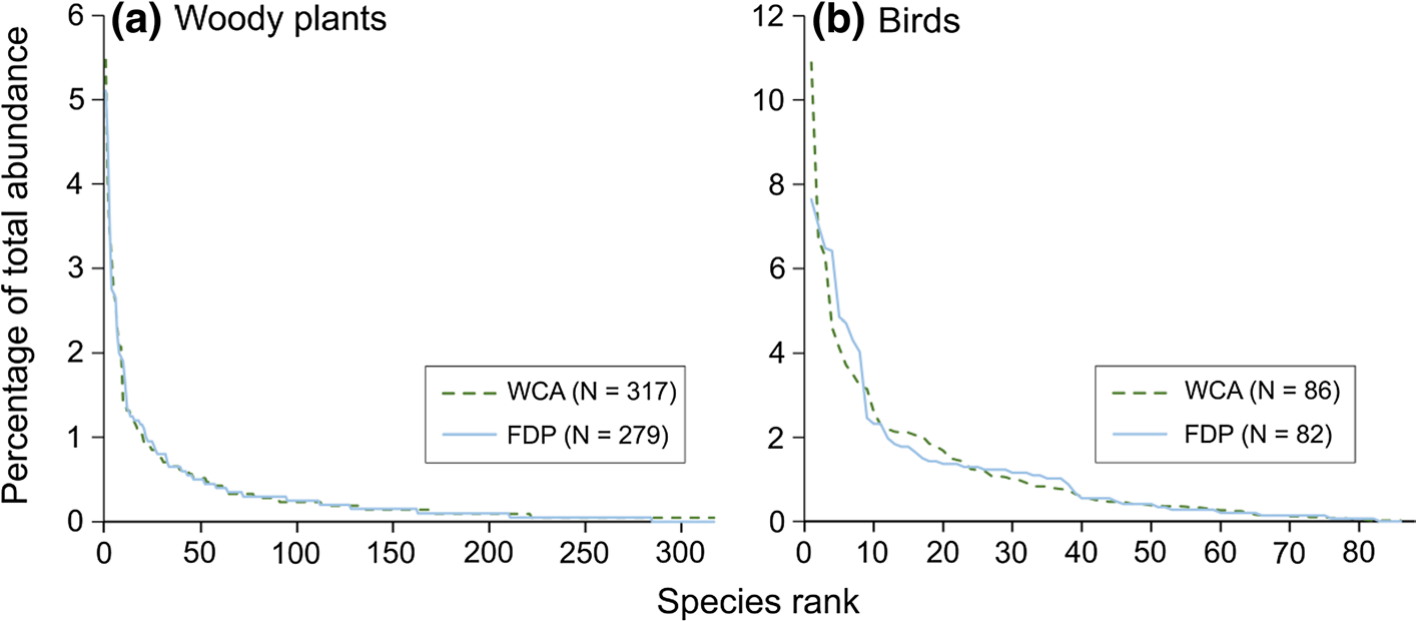
Rank dominance for **a** woody plants and **b** birds in the WCA (dashed lines) and FDP (solid lines). Curves show the percentage of all individuals represented by each species (*N* = 317 and 279 woody plant species for WCA and FDP, respectively; *N* = 86 and 82 bird species for WCA and FDP, respectively)

We recorded a total of 4060 individual woody plants ≥ 5 cm DBH, representing 379 species, across both surveys. A total of 2119 individual woody plants ≥ 5 cm DBH were recorded across the 43 sample locations of the WCA survey, representing a total of 317 species. The 40 plots taken from the FDP survey contained 1941 individual woody plants ≥ 5 cm DBH from 279 species, i.e. 88% of WCA species richness. A total of 217 species (57.3%) were present in both surveys. 68.5% of species (comprising 90% of abundance) found in the WCA were also present in the FDP, while 77.8% of species (comprising 94.1% of abundance) found in the FDP were present in the WCA. The rarefaction curves from both surveys do not appear to approach an asymptote, suggesting greater sampling effort is necessary to achieve accurate species richness estimates (Fig. 2). Nevertheless, the Chao2 richness indicator estimated species richness values of 419.8 (WCA) and 353.1 (FDP). From observed and extrapolated species richness (Fig. 2) a clear separation can be observed between species rarefaction curves for the two surveys. The lack of an overlap of 95% confidence intervals for the plant data indicates a significantly higher species richness across the WCA than in the FDP. However, despite the difference in species richness, woody plants from the two surveys show very similar dominance structure patterns (Fig. 3).

The results described above are supported by a comparison of the WCA dataset (40 plots) and the full FDP dataset (1250 plots). The total species richness (452 species) in the latter dataset covering the entirety of the FDP is higher due to a strikingly more intense sampling effort. Nevertheless, the WCA dataset still comprises 53 (16.7%) unique species (7.3% of individuals) that did not occur in the full FDP dataset.

### Species richness of bird community

We recorded a total of 6389 individual birds of 93 species across both surveys. This included 4976 individuals of 86 species from the 320 point counts in the WCA and 1420 individuals of 82 species (i.e. 95.3% of WCA species richness) from the 169 point counts in the FDP. Community composition was similar between the two surveys. A total of 79 species (84.9%) were present in both surveys. 91.9% of species (representing 99% of abundance) from the WCA were also present in the FDP, while 96.3% of species (representing 99.6% of abundance) found in the FDP were present in the WCA. In contrast to plants, rarefaction curves showed no significant difference between the two datasets (95% CI) for 169 point counts, the highest sample size available for both WCA and FDP (Fig. 2). This overlap persisted even when using 84% confidence intervals (Fig. S1), a technique which has been shown to robustly mimic 0.05 pairwise statistical tests when comparing species richness values (MacGregor-Fors and Payton 2013). Moreover, unlike plants, rarefaction curves for both bird survey datasets closely approach an asymptote (Fig. 2). Extrapolation using the Chao2 richness indicator produced estimated asymptotic species richness values of 89.3 (WCA) and 83.9 (FDP). Species rank abundance curves (Fig. 3) are similar in shape for the two surveys, although the most common species represented a higher proportion of records at WCA (*Pitohui kirhocephalus*, 10.9% of records) than the FDP (*Meliphaga* sp., 7.6%).

### Beta-diversity

**Fig. 4:**
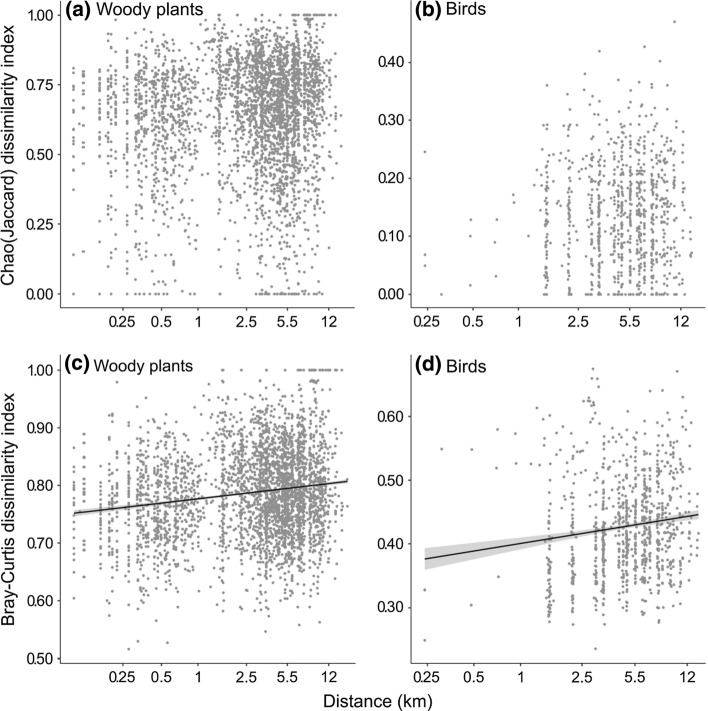
Pairwise relationships of beta-diversity indices and geographical distances between sampling sites, shown for woody plants (**a**, **c**) and birds (**b**, **d**). We used two dissimilarity indices: Chao-Jaccard (**a**, **b**) and Bray–Curtis (**c**, **d**). Linear approximation with shaded area representing standard error was used for significant relationships (Pearson *r*, Mantel test, *p* < 0.05)

We did not find any relationship between pairwise distance and dissimilarity in woody plant community composition when measured using the Chao-Jaccard dissimilarity index (Fig. 4a; Mantel test, 9999 permutations, *r* = 0.089, *p* = 0.069). However, when using the Bray–Curtis index, the dissimilarity of woody plant communities significantly increased with geographic distance (Fig. 4c; Mantel test, 9999 permutations, Mantel *r* = 0.135, *p* = 0.034). Similarly, when using the Chao-Jaccard dissimilarity estimator, we did not detect a significant relationship between bird community dissimilarity and inter-site distance across the pooled WCA/FDP sample locations (Fig. 4b; Mantel test, 9999 permutations, Mantel *r* = 0.033, *p* = 0.296). In contrast, beta-diversity with the Bray–Curtis index did show a positive relationship between community dissimilarity and distance (Fig. 4d; Mantel test, 9999 permutations, *r* = 0.132, *p* = 0.028).

Dissimilarities (Chao-Jaccard; Bray–Curtis) between sampling locations located within the smaller study area (FDP) were on average lower than those within the larger WCA both for both plants and birds (Fig. S2).

### Effect of environmental variables

**Fig. 5:**
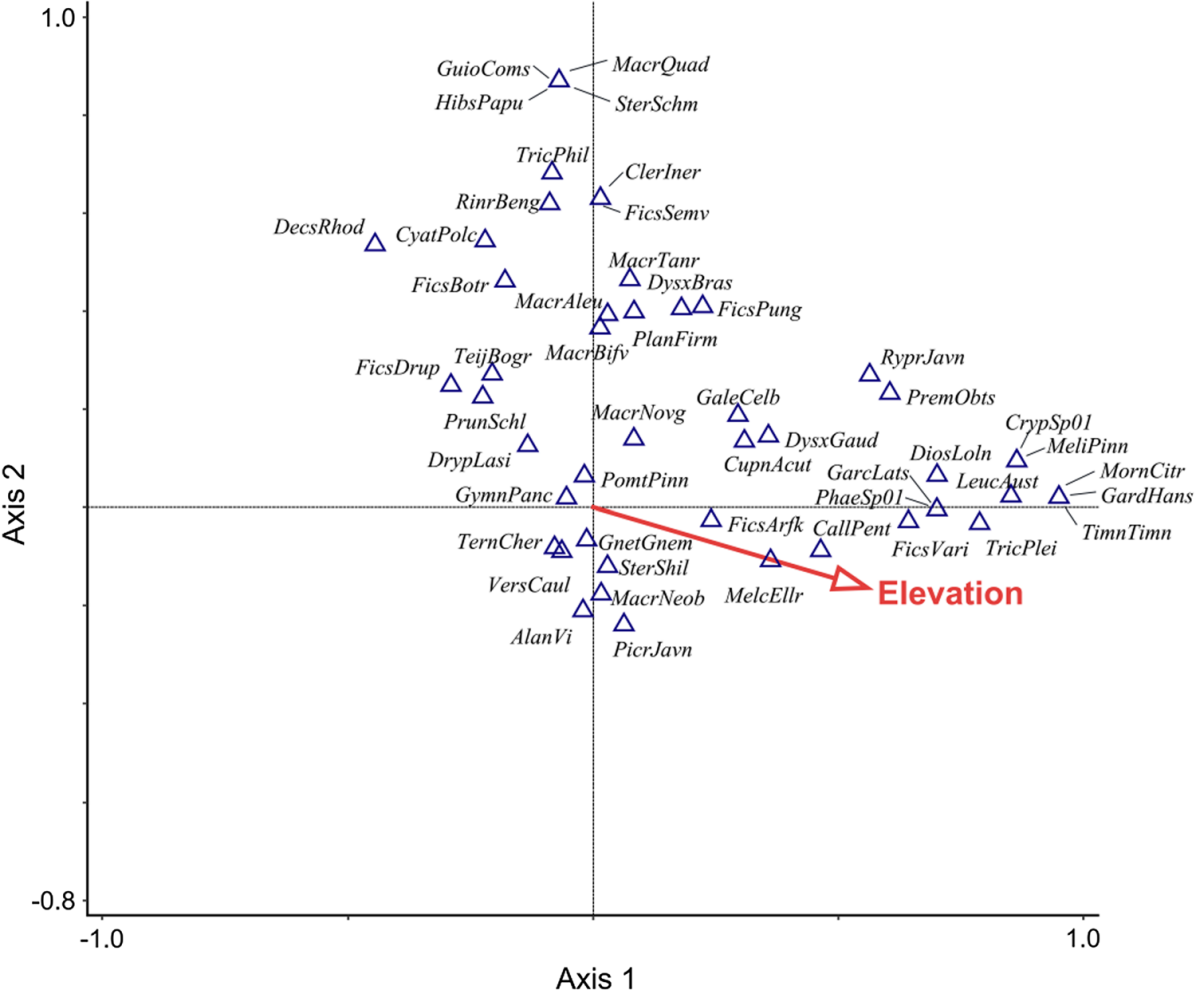
Canonical Correspondence Analysis (CCA) ordination diagram showing composition of woody plants (centroids with plant species codes) and influence of elevation as a primary predictor after the effect of space was filtered out (total variation = 10.079, axis 1 eigenvalue = 0.236, axis 2 eigenvalue = 0.195). The diagram displays 50 species with the highest fit value. Corresponding full species names are provided in Table S2

A significant proportion of variation in woody plant species composition was explained by elevation of sampling locations (Fig. 5, Table S1; CCA, *F* = 1.6, *p* = 0.001, 22% of explained variation). Spatial structure of sampling locations, however, accounted for larger proportion of explained variation (77.1%). The primary (elevation) and space predictors shared 0.9% of explained variation and together they explained 9% of the total variation.

**Fig. 6:**
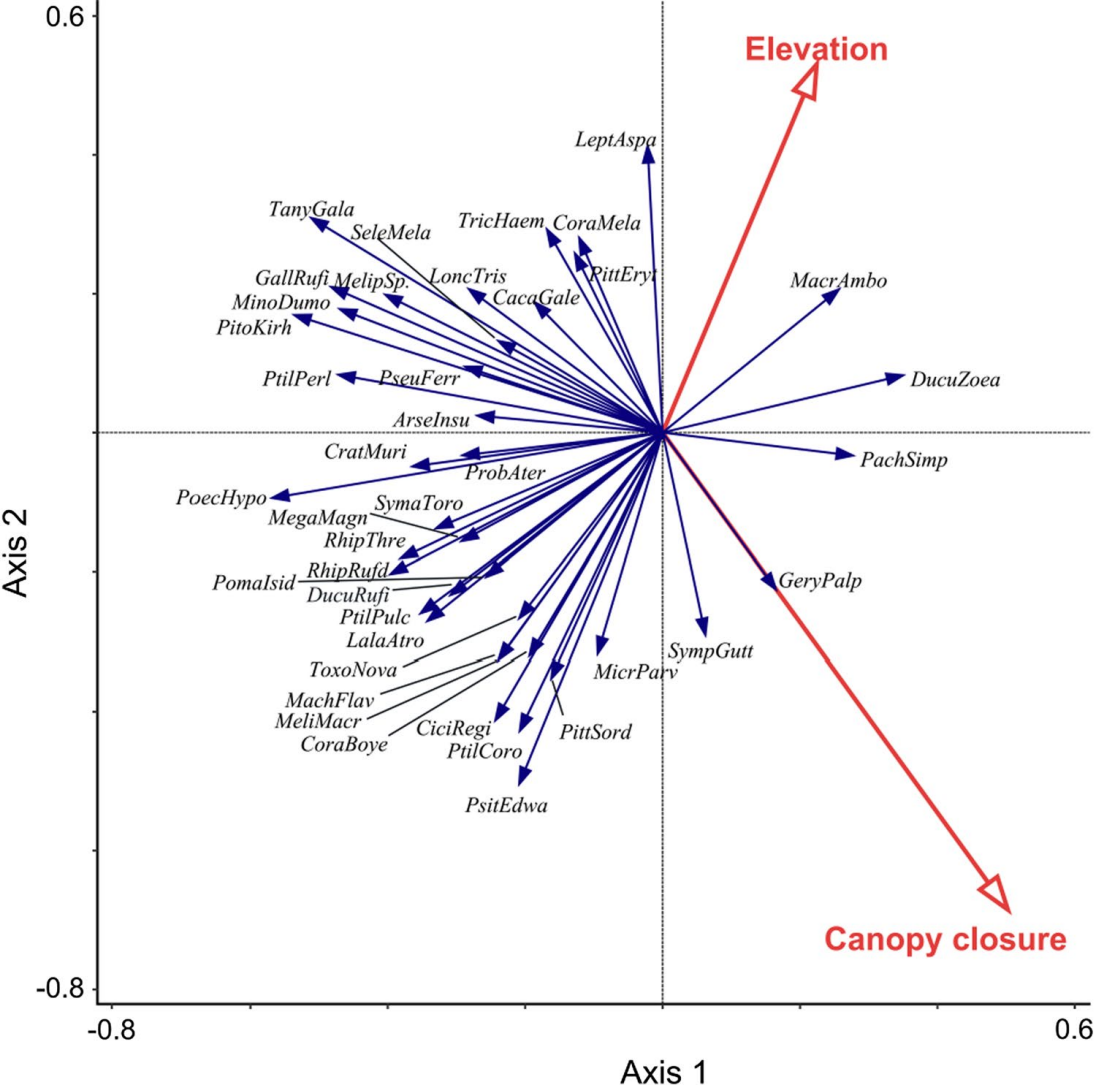
Redundancy Analysis (RDA) ordination diagram showing composition of bird community (species arrows) in relation to elevation and canopy closure after the effect of space was filtered out (total variation = 9792.023, axis 1 eigenvalue = 0.121, axis 2 eigenvalue = 0.042). The 40 species with the highest fit value are displayed. Corresponding full species names are provided in Table S3

Forward selection found canopy closure, number of individual trees within plots and elevation to be the significant primary predictors of bird community composition across the pooled datasets (Fig. 6, Table S1; RDA, canopy closure: *F* = 2.6, *p* = 0.009; number of trees: *F* = 2.0, *p* = 0.013; elevation: *F* = 2.0, *p* = 0.028). The primary predictors accounted for 40.3% of explained variation, whereas space accounted for 51.0% of explained variation (8.7% of explained variation was shared between primary and space predictors). All predictors explained 29.9% of the total variation.

## Discussion

The scale-dependent nature of diversity patterns has long been recognized in the field of ecology (Arrhenius 1921; Connor and McCoy 1979; Shmida and Wilson 1985; Huston 1999; Gaston 2000; Rahbek 2005). Studies exploring this relationship tend to draw a distinction between “local” diversity, determined mostly by species niche differentiation and direct inter-specific interactions, and “regional” diversity determined by species pools and evolutionary dynamics. However, few studies have focussed on the species diversity patterns on spatial scales between these two extremes, within tens to thousands of km^2^ within relatively uniform habitats. This study relates diversity patterns of woody plants and birds on this intermediate scale, represented here by 100 km^2^ of a lowland rainforest, to the local patterns within a 50 ha forest plot.

The magnitude of bird species diversity and the rate of species accumulation with increasing sample size within the Wanang FDP was almost perfectly mirrored by that within the 10,000 ha WCA. The overlap in species composition between the data sets from 50 and 10,000 ha was also very high, suggesting that for bird diversity the FDP is representative of the wider area. Conversely, plant species richness was shown to be significantly higher across the WCA than within the selected plots from the FDP, although the 50 ha diversity still represented 88% of the species richness within 10,000 ha. Thus any hierarchical structure between WCA and FDP appears non-existent for birds and weak for plants.

Bird species composition did not show any trends over the 10,000 ha area, i.e. there was no increase in Chao-Jaccard dissimilarity with increasing distance between the compared pairs of samples. However, there was a slow but significant change in the community composition taking account of species abundances with distance. This pattern, in combination with the relatively large proportion of variability in species composition explained by environmental variables, suggests that most of the variability in bird communities is fine-grained, within 1 km distances and in response to vegetation structure and terrain. The contrasting results of the two indices suggest that any variability over the scale explored in this study is primarily driven by relatively few common species. Dissimilarity in woody plant species composition and community composition showed similar patterns to those of birds. Woody plants displayed a small, but non-significant (*p* = 0.069), increase in species dissimilarity with distance, while community dissimilarity accounting for species abundances was significant and similar to that of birds. In addition, when comparing the two datasets separately plants showed higher mean dissimilarity across the wider WCA than in the FDP for both indices, indicating higher variation in plant community and species composition at the intermediate than at the local scale. Such a result is in line with the expectation that variance between samples of a constant grain size should increase with increasing sample extent (Wiens 1989). Birds showed a similar pattern between datasets for both indices, although displaying lower overall dissimilarity than plants in all cases.

The difference in compositional dissimilarity patterns between plants and birds, while subtle, may be attributable to different ecological processes acting on the two taxa at the scales explored in this study. It is posited that dispersal limitation is a significant causal factor of species aggregation in tropical forest trees (Seidler and Plotkin 2006; Myers et al. 2013), with non-animal dispersed tree species likely to be dispersal-limited across spatial scales (Seidler and Plotkin 2006; Beaudrot et al. 2013). Evidence from tropical forest dynamics plots points to abiotic environmental filtering also being an important driver of tropical tree species distributions, at least at small (< 1 km) scales (Plotkin et al. 2002). Our results suggest that distance between sampling locations may account for a relatively large proportion of variability in woody plant species composition when compared with environmental characteristics. However, because we only used elevation as an environmental predictor, these results should be interpreted with caution. As a highly mobile taxon, birds are less restricted in their dispersal than plants (Soininen et al. 2007), especially in unfragmented lowland habitats such as the one studied here (Van Houtan et al. 2007). Although the results of this study suggest bird species composition is related to habitat structure at fine scales, we found very little variation across the (relatively environmentally homogenous) broader scale of the WCA. Taken together, these observations suggest that dispersal limitation, perhaps driven primarily by limited dispersal of non-fleshy fruited (i.e. non-bird dispersed) trees, is a key factor in explaining the distribution differences observed between the two taxa. However, a current lack of studies on lowland rainforest beta-diversity at this scale limits scope for comparative assessment.

On comparing two separate 100 ha plots located within a 650 ha reserve in the Ecuadorian Amazon, Blake (2007) found bird species composition to be almost identical between plots, the only major variation being in individual species’ distributions and abundances, reflecting small-scale differences in habitat structure and availability between plots. In a broader analysis of plots across French Guiana, Thiollay (2002) found that despite having sparse local populations, the vast majority of bird species had wide range sizes, thus masking any general determinant of community structure. Species turnover between sites was found to be 29% on average, for inter-site distances 15–320 km, i.e. far higher than the distances analysed in this study.

In an analysis of plant beta-diversity from sites across Panama, Ecuador and Peru, Condit et al. (2002) showed that in the range of pairwise distances represented in this study (approximately 0–15 km), percentage of shared plant species between plots decreased in all three regions. In both Ecuador and Peru, this decrease tended to level off beyond around 20 km, suggesting that in these cases local- and intermediate-scale variation plays a more important role in determining plant community composition than broader-scale patterns. In a tropical dry forest in southern Mexico, Gallardo-Cruz et al. (2010) demonstrated a similar pattern of decreasing plant community similarity with increasing distance, in this case within a range of 0–6 km. In a Malaysian lowland rainforest, the extent to which a 50 ha ForestGEO plot represented the wider peninsular flora was found to vary dramatically among plant families, with representativeness within large families ranging between 5 and 66% (LaFrankie 1996).

The results of this study and those described above highlight the varying importance of so-called intermediate-scale patterns in determining overall community composition for different taxa, and therefore in determining optimal sampling regimes. The relative homogeneity of bird communities across the WCA suggests that bird species richness and community composition across 10,000 ha of lowland rainforest may be accurately estimated by sampling within a 50 ha plot. This result is particularly notable given the lack of previous studies on birds in ForestGEO plots. For plants however, surveys that are limited to 50 ha may miss a significant proportion of diversity due to the higher influence of local-scale variation on overall community composition in woody plants. Nevertheless, the observed slow distance decay of both woody plants and birds across the WCA provides support for the use of the ForestGEO Plot as a means of representing wider biodiversity for both taxa. Extending the study of intermediate-scale diversity patterns to important rainforest taxa such as insects will be necessary if we are to gain a fuller picture of biodiversity across spatial scales in lowland rainforests.

## Supplementary Information

Below is the link to the electronic supplementary material.

Supplementary file1 (DOCX 1684 kb)

## Data Availability

The datasets generated during the current study will be made publicly available in the Dryad repository, upon publication of the manuscript.

## Declarations

### Statement of Legality

All fieldwork activities complied with the current laws of Papua New Guinea.
