# Supplementary material for: Spatial scaling of plant and bird diversity from 50 to 10,000 ha in a lowland tropical rainforest
Source: Oecologia. 2021 May 5;196(1):101–13. doi: 10.1007/s00442-021-04925-8 (PMC8139934; doi:10.1007/s00442-021-04925-8)
Supplement: Supplementary file 1 — Supplementary file1 (DOCX 1684 kb) [file 442_2021_4925_MOESM1_ESM.docx]

Supplementary Material**: Spatial scaling of plant and bird diversity from 50 to 10,000 ha in a lowland tropical rainforest**

Authors: Richard J. Hazell, Kryštof Chmel, Jan Riegert, Luda Paul, Brus Isua, Graham S. Kaina, Pavel Fibich, Kenneth Molem, Alan J. A. Stewart, Mika R. Peck, George D. Weiblen, Vojtech Novotny

Corresponding Author: Richard J. Hazell

Department of Evolution, Behaviour and Environment, School of Life Sciences, University of Sussex, Brighton BN1 9QG, United Kingdom

Email: richard.hazell@hotmail.co.uk; r.j.hazell@sussex.ac.uk

Phone: +44 (0)7817646804


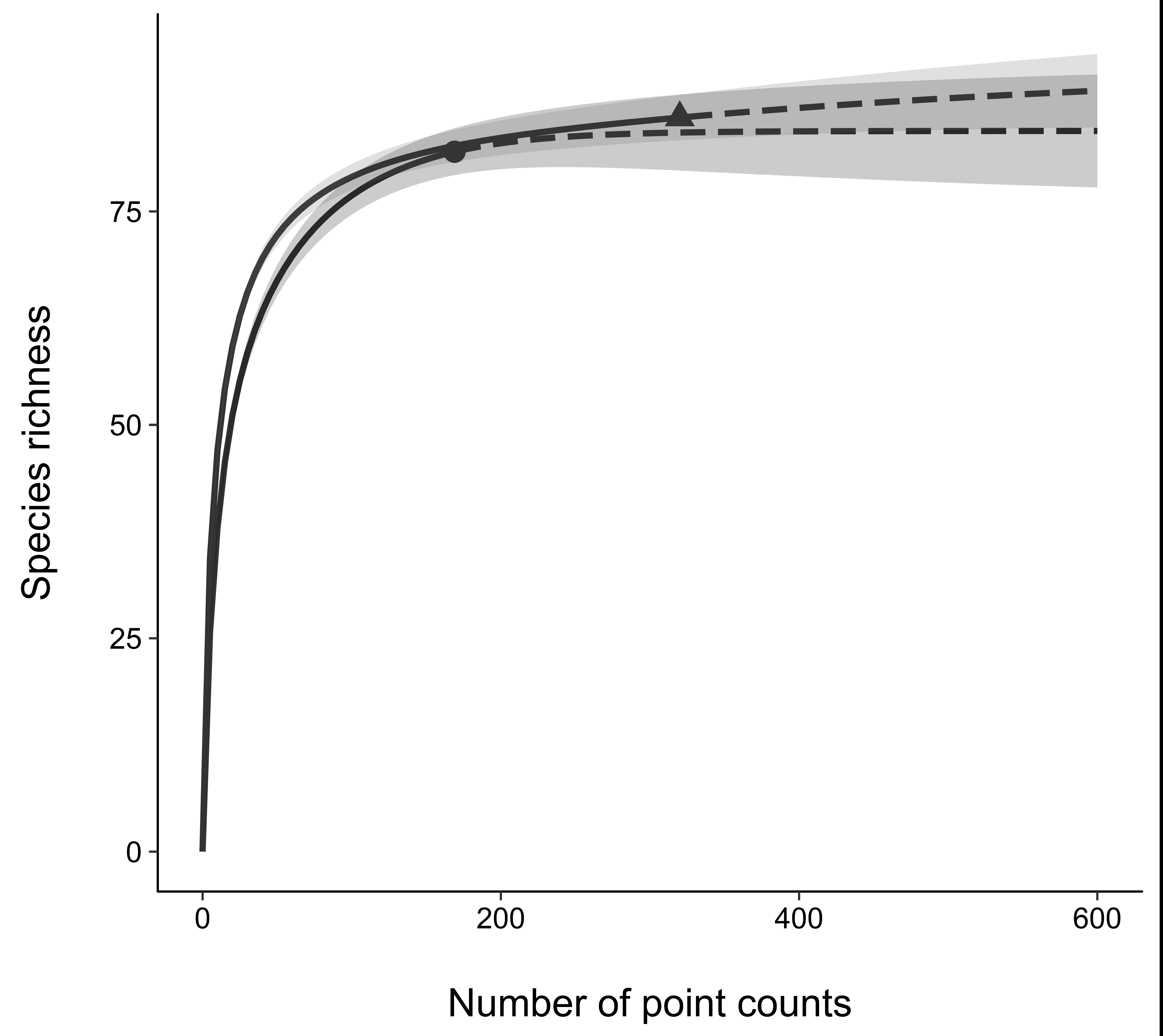


**Figure S1.** Bird species richness as shown in Figure 2, in this case represented by rarefaction curves for WCA (black triangle) and FDP (black circle) with ±84% confidence intervals (shaded areas). Solid lines show interpolated rarefaction curves. Dashed lines represent extrapolated rarefactions exceeding our sampling effort. Species accumulation was calculated by site (individual point count location) for a total of 320 (WCA) and 169 (FDP) sites.


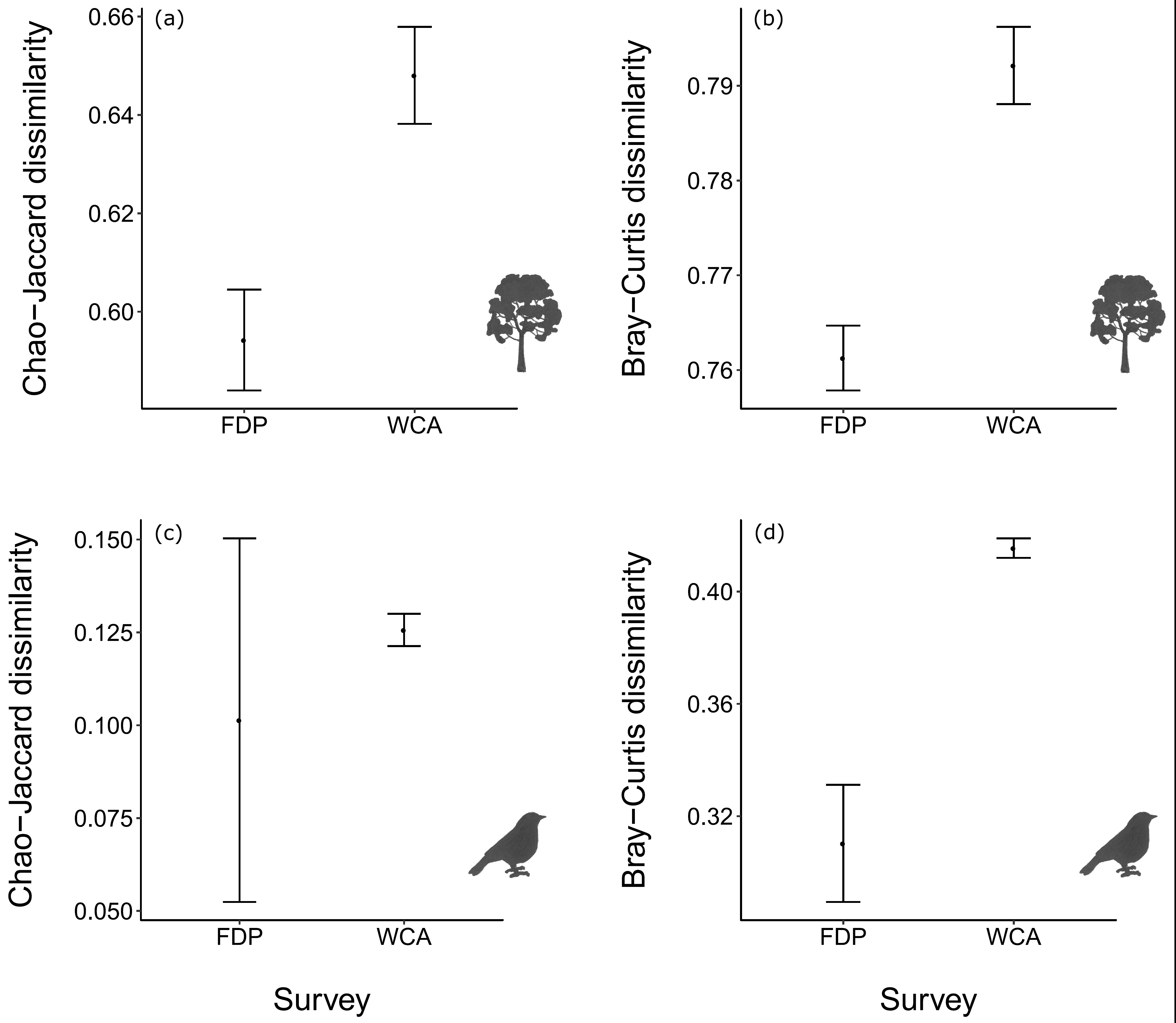


**Figure S2.** Beta-diversity of woody plants (a,b) and birds (c,d) within FDP and WCA surveys. Mean Chao-Jaccard (a, c) and Bray-Curtis dissimilarity (b, d) distances with error bars representing 0.95 confidence intervals are shown. Between surveys differences in dissimilarity (Permutational MANOVA, 999 permutations): (a) pseudo-F_1560,1806_ = 5.28, P = 0.001; (b) pseudo-F_1560,1806_ = 2.95, P = 0.001; (c) pseudo-F_12,1560_ = 5.89, P = 0.006; (d) pseudo-F_1560,1806_ = 5.16, P = 0.001.

**Table S1.** Description of results displayed in woody plant canonical correspondence analysis (CCA) and bird redundancy analysis (RDA) ordination diagrams (Figures 5 and 6 respectively).

| **Analysis** | **Description** |
| --- | --- |
| Woody plant CCA ordination (Figure 5) | Elevation was positively correlated with the first ordination axis  (correlation coefficient = 0.43) and negatively correlated with the  second ordination axis (-0.36). Species optima of several woody  plant species (e.g. *Callicarpa pentandra*, *Ficus arfakensis* and  *Melicope elleryana*) were found in plots located at higher  elevations, i.e. on ridges. Other species (e.g. *Drypetes  lasiogynoides*, *Ficus drupacea*, *Pometia pinnata* and *Prunus  schlechteri*) responded negatively to elevation and occurred more  in valleys. |
| Bird RDA ordination (Figure 6) | Canopy closure was positively correlated with the first ordination  axis (correlation coefficient 0.47) and negatively with the second  ordination axis (-0.56). Elevation was positively correlated with the second ordination axis (0.36) and less so with first ordination axis (0.25). Common species (e.g. *Coracina boyeri*, *Cicinnurus regius* and *Ptilinopus coronulatus*) had highest abundances in sampling locations of lower elevation, while *Leptocoma aspasia* and *Macropygia amboinensis* tended to be more abundant at higher elevations. Simultaneously, the analysis identified species that avoided dense sites with high canopy closure (e.g. *Cacatua gallerita*, *Leptocoma aspasia*, *Tanysiptera galatea* and *Trichoglossus haematodus*). The only species that preferred sites with high canopy closure were *Gerygone palpebrosa* and *Symposiachrus guttula*. |

**Table S2.** Woody plant species names corresponding to species codes displayed in Figure 5. Morphospecies are presented in the cases of *Cryptocarya sp.01* and *Phaeanthus sp.01* where identification to species level was not possible.

| Species Code | Species Name |
| --- | --- |
| AlanVi | *Alangium villosum* |
| CallPent | *Callicarpa pentandra* |
| ClerIner | *Clerodendrum inerme* |
| CrypSp01 | *Cryptocarya sp.01* |
| CupnAcut | *Cupaniopsis acuticarpa* |
| CyatPolc | *Cyathocalyx polycarpa* |
| DecsRhod | *Decaspermum rhodoleucum* |
| DiosLoln | *Diospyros lolin* |
| DrypLasi | *Drypetes lasiogynoides* |
| DysxBras | *Dysoxylum brassii* |
| DysxGaud | *Dysoxylum gaudichaudianum* |
| FicsArfk | *Ficus arfakensis* |
| FicsBotr | *Ficus botryocarpa* |
| FicsDrup | *Ficus drupacea* |
| FicsPung | *Ficus pungens* |
| FicsSemv | *Ficus semivestita* |
| FicsVari | *Ficus variegate* |
| GaleCelb | *Galearia celebica* |
| GarcLats | *Garcinia latissimi* |
| GardHans | *Gardenia hansemannii* |
| GnetGnem | *Gnetum gnemon* |
| GuioComs | *Guioa comesperma* |
| GymnPanc | *Gymnacranthera paniculata* |
| HibsPapu | *Hibiscus papuadendron* |
| LeucAust | *Leucosyke australis* |
| MacrAleu | *Macaranga aleuritoides* |
| MacrBifv | *Macaranga bifoveata* |
| MacrNeob | *Macaranga neobritannica* |
| MacrNovg | *Macaranga novoguineensis* |
| MacrQuad | *Macaranga quadriglandulosa* |
| MacrTanr | *Macaranga tanarius* |
| MelcEllr | *Melicope elleryana* |
| MeliPinn | *Meliosma pinnata* |
| MornCitr | *Morinda citrifolia* |
| PhaeSp01 | *Phaeanthus sp.01* |
| PicrJavn | *Picrasma javanica* |
| PlanFirm | *Planchonella firma* |
| PomtPinn | *Pometia pinnata* |
| PremObts | *Premna obtusifolia* |
| PrunSchl | *Prunus schlechteri* |
| RinrBeng | *Rinorea bengalensis* |
| RyprJavn | *Ryparosa javanica* |
| SterShil | *Sterculia shillinglawii* |
| SterSchm | *Sterculia schumanniana* |
| TeijBogr | *Teijsmanniodendron bogoriense* |
| TernCher | *Ternstroemia cherry* |
| TimnTimn | *Timonius timon* |
| TricPhil | *Trichadenia philippinensis* |
| TricPlei | *Trichospermum pleiostigma* |
| VersCaul | *Versteegia cauliflora* |

**Table S3.** Bird species names corresponding to species codes displayed in Figure 6. Two species from the genus *Meliphaga* are presented in the case of *MeliSp.* where it was usually not possible to distinguish between the two species.

| Species Code | Species Name |
| --- | --- |
| ArseInsu | *Arses insularis* |
| CacaGale | *Cacatua galerita* |
| CiciRegi | *Cicinnurus regius* |
| CoraBoye | *Coracina boyeri* |
| CoraMela | *Coracina melas* |
| CratMuri | *Crateroscelis murina* |
| DucuRufi | *Ducula rufigaster* |
| DucuZoea | *Ducula zoeae* |
| GallRufi | *Gallicolumba rufigula* |
| GeryPalp | *Gerygone palpebrosa* |
| LalaAtro | *Lalage atrovirens* |
| LeptAspa | *Leptocoma Aspasia* |
| LoncTris | *Lonchura tristissima* |
| MacrAmbo | *Macropygia amboinensis* |
| MachFlav | *Machaerirhynchus flaviventer* |
| MegaMagn | *Megaloprepia magnifica* |
| MeliMacr | *Melidora macrorrhina* |
| MeliSp. | *Meliphaga analoga / M. Aruensis* |
| MicrParv | *Microdynamis parva* |
| MinoDumo | *Mino dumontii* |
| PachSimp | *Pachycephala simplex* |
| PitoKirh | *Pitohui kirhocephalus* |
| PittEryt | *Pitta erythrogaster* |
| PittSord | *Pitta sordida* |
| PoecHypo | *Poecilodryas hypoleuca* |
| PomaIsid | *Pomatostomus isidorei* |
| ProbAter | *Probosciger aterrimus* |
| PseuFerr | *Pseudorectes ferrugineus* |
| PsitEdwa | *Psittaculirostris edwardsii* |
| PtilCoro | *Ptilinopus coronulatus* |
| PtilPerl | *Ptilinopus perlatus* |
| PtilPulc | *Ptilinopus pulchellus* |
| RhipRufd | *Rhipidura rufidorsa* |
| RhipThre | *Rhipidura threnothorax* |
| SeleMela | *Seleucidis melanoleucus* |
| SymaToro | *Syma torotoro* |
| SympGutt | *Symposiachrus guttula* |
| TanyGala | *Tanysiptera galatea* |
| ToxoNova | *Toxorhamphus novaeguineae* |
| TricHaem | *Trichoglossus haematodus* |
